# Comparative Prognostic Analysis of the Tall Cell Subtype of Papillary Thyroid Carcinoma With the Conventional Subtype Following the WHO 2022 Revision: An Indian Cohort Study

**DOI:** 10.7759/cureus.98485

**Published:** 2025-12-04

**Authors:** Jameera Nazer, Smitha NV, Usha Menon, Krishnakumar Thankappan

**Affiliations:** 1 Pathology, Amrita Institute of Medical Sciences, Kochi, IND; 2 Endocrinology, Amrita Institute of Medical Sciences, Kochi, IND; 3 Head and Neck Surgery, Amrita Institute of Medical Sciences, Kochi, IND

**Keywords:** endocrine oncology, head &neck pathology, papillary carcinoma of thyroid, tall cell, thyroid pathology

## Abstract

​​​Introduction: Papillary thyroid carcinoma (PTC) is the most common thyroid cancer with multiple subtypes and accounts for the majority of thyroid malignancies worldwide. The tall cell subtype (TC-PTC) is recognised for its aggressive behaviour, poorer prognosis, and higher likelihood of extrathyroidal extension and distant metastasis. This study aimed to evaluate the prognostic validity of the revised WHO 2022 criteria for TC-PTC (≥30% of cells at least three times as tall as wide with dense eosinophilic cytoplasm and distinct cell membranes), compare clinicopathological features and outcomes of PTCs with any tall cell features (PTC-TCF) with classical PTC (cPTC), and determine whether less stringent morphological thresholds better identify clinically aggressive tumours.

Methods: This retrospective comparative study was conducted in the Pathology Department of a tertiary care hospital in Kerala over four years (2015-2019) with a minimum five-year follow-up. Two groups were compared: PTC-TCF and cPTC, designated as cases and controls respectively. Based on distant metastasis proportions in TC-PTC and cPTC from an earlier study, with 95% confidence, 80% power, and a 1:4 ratio, the minimum required sample size was 66 cases and 264 controls (total of 330). Clinical and histopathological details were obtained from electronic medical records, and follow-up data from the institutional cancer registry. Archived H&E slides of cases with tall cell components were retrieved and reassessed for degree and percentage of tall cells. Statistical analysis was performed using IBM SPSS version 20.0 (IBM Corp., Armonk, NY, USA), with p < 0.05 considered statistically significant.

Results: Of the 330 cases studied, 66 (20%) showed tall cell features. Compared with cPTC, PTC-TCF cases demonstrated significantly higher rates of extrathyroidal extension (ETE) (p < 0.001), advanced pathological T stage (p < 0.001), distant metastasis at presentation (p = 0.005), recurrence (p < 0.001), and mortality (p = 0.020). On multivariate analysis adjusted for tumour size and nodal status, T2 (HR = 12.70, p < 0.001) and T3 (HR = 17.40, p < 0.001) stages retained independent statistical significance.

Tumour subtype (p < 0.001), tumour size (p = 0.043), modified American Thyroid Association (ATA) risk class (p = 0.006), proportion of cells with height twice as tall as width (2x) ≥ 30% (p = 0.003), T stage (p = 0.043), and M stage at presentation (p = 0.021) were found significant with respect to recurrence and death trends on univariate analysis. Categorising tumours using the WHO 2022 definition for tall cell subtype (≥ 30% of cells with height thrice as tall as width or 3x) did not yield significant correlation with recurrence or mortality rates (p = 0.197). After controlling for tumour focality, ETE, and lymphovascular invasion (LVI), presence of 2× tall cells in ≥ 30% retained significance (HR = 2.46, p = 0.048) with respect to prognostic outcomes on multivariate analysis.

Conclusion: In our study, the proportion of 2x tall cells with a cutoff of 30% showed statistical significance with respect to recurrence and mortality, which was retained even on multivariate analysis. This indicates that setting a criterion of 2x-3x tall cells for the diagnosis of tall cell PTC could better predict the prognosis.

## Introduction

The tall cell subtype of papillary thyroid carcinoma (TC-PTC) is well known for its more aggressive behaviour and worse prognosis compared to the classic type (cPTC). It is often associated with higher age at diagnosis, larger tumour size, higher likelihood of extrathyroidal extension, and greater risk of distant metastasis. Due to its aggressive nature, tall cell subtype of papillary thyroid carcinoma is classified as an intermediate or high-risk differentiated thyroid carcinoma (DTC) in the American Thyroid Association (ATA) risk grouping and typically requires a more intensive treatment approach and careful long-term follow-up [1].

Despite the acknowledged prognostic significance of TC-PTC, pathologists have yet to establish a consensus on diagnostic criteria in clinical practice. The evolution of TC-PTC definitions over time reflects this lack of agreement, with various definitions being used by different practitioners. Over time, multiple studies have used either a 2:1 or 3:1 height-to-width ratio and differing cutoffs for the proportion of tall cells required. This has contributed to diagnostic inconsistency and challenges in assessing the prognostic impact of this subtype [2,3]. 

The 2017 WHO criteria defined TC-PTC as comprising over 30% of cells that are two or three times taller than they are wide [2]. In 2022, the WHO revised these criteria, requiring cells to be at least thrice as tall as they are wide, showing dense eosinophilic cytoplasm and distinct cell membranes [4]. Studies worldwide suggest that TC-PTC is underdiagnosed; reviews of cases initially diagnosed as classic PTC found that 1-13% were actually TC-PTC [5]. 

In addition to the height-to-width ratio and percentage criteria, TC-PTC diagnosis also requires the use of additional soft histological diagnostic criteria. Prominent/exaggerated nuclear characteristics of PTC, eosinophilic cytoplasm with defined cell boundaries, and elongated "tram-track" follicles are the criteria endorsed by the WHO in 2022 [4]. To distinguish TC-PTC from the oncocytic subtype, which is not more aggressive than cPTC, these characteristics are crucial. Frequently, the cells of oncocytic subtype lack the clear cell boundaries or elongated "tram track" follicular growth patterns inherent to TC-PTC. They also frequently have a more strongly granular eosinophilic cytoplasm and are not typically two to three times as tall as they are wide [3]. 

Given the ongoing debate regarding the diagnostic thresholds and prognostic significance of tall cell morphology in PTC, further clarification of the diagnostic criteria is essential for accurate risk stratification. The present study aims to evaluate the prognostic relevance of the 2022 WHO criteria for TC-PTC and to compare the clinicopathological characteristics and long-term outcomes of PTC with any amount of tall cell features (PTC-TCF) against those of cPTCs. In doing so, we seek to determine whether a less stringent criteria - such as a 2× to 3x height-to-width ratio, or lower proportion cutoff along with soft histological diagnostic features, may better capture the clinically aggressive tumours than by the current WHO definition.

Strengths of the study

All cases included in the study were derived from patients attending a single referral centre, specifically a dedicated thyroid clinic that operates under standardized management protocols in accordance with the ATA criteria for risk stratification and follow-up. Owing to the implementation of uniform synoptic reporting in our department, all essential histopathological parameters required for evaluation were readily accessible through the electronic medical record system.

For each case of PTC-TCF included in the study, an average of 5.8 slides (ranging from 1 to 20 slides) were examined. The tumour sizes ranged from 1 to 6 cm, with a mean size of 3.6 cm. This extensive slide evaluation is expected to provide a more accurate estimation of the proportion of tall cells compared to earlier studies, which typically assessed only a single slide per case.

Limitations of the study

The sample size in the present study was relatively small compared to other similar studies. The follow-up period was also comparatively shorter, with an average duration of 94 months.

A total of 99 cases (30%) had missing information for one or more clinicopathological or follow-up parameters. Specifically, 85 (32%) cases in the cPTC group and 14 cases (21.2%) in the PTC-TCF group lacked data on either tumour focality, extrathyroidal extension (ETE), lymphovascular invasion (LVI), or number of lymph nodes involved. Although these were second-opinion referrals from external centres that did not follow our synoptic reporting format, our analysis still covered an average of 5.8 slides per case.

To assess the effect of missing data, sensitivity analyses were performed. Excluding these incomplete cases did not materially alter the overall results. The associations between tumour type and recurrence (p < 0.001) as well as mortality (p = 0.020) remained statistically significant. Hazard ratios in the multivariate Cox regression analysis remained stable with regards to the pathological T stage (HR for T2 = 12.70, 95% CI: 6.34-25.42; p < 0.001; HR for T3 = 17.40, 95% CI: 9.12-33.18; p < 0.001), and the proportion of 2x tall cells ≥ 30% continued to be an independent predictor of adverse outcomes on multivariate analysis (HR = 2.46; p = 0.048). Thus, while the missing data modestly affected completeness, it did not significantly impact the statistical robustness or direction of the findings.

Twenty-five of the total 330 patients (7.6 %) could not be contacted for follow-up. These cases were classified as “lost to follow-up” and were excluded from outcome analysis.

The retrospective design inherently limits control over confounding variables and introduces possible selection and information bias. Reliance on existing records and archived materials also prevented the uniform capture of some variables.

Although slides were independently reviewed by two pathologists, a formal interobserver concordance (Kappa) analysis was not performed, which limits reproducibility of results in tall cell quantification. Discrepancies were resolved through consensus review to minimize bias.

As a single-centre study from a South Indian tertiary institution, findings may not be fully generalizable to broader populations or to centres using differing diagnostic or management practices. Larger multicentric and prospective studies are needed to confirm these results across varied cohorts.

## Materials and methods

This is a retrospective comparative study conducted in the department of pathology in a tertiary centre in Kerala, India. PTCs diagnosed in our centre over a period of four years (2015 July 1st to 2019 June 30th) with a minimum of five-year follow-up, were selected from the archives of the pathology department and thyroid clinic. 

The study compares two groups - PTC-TCF cases and PTC cases with pure conventional morphology (cPTC). Based on the proportion of distant metastasis among TC-PTC (20%) and cPTC (6.5%) as obtained from an earlier publication by Sampathkumar et al., with a 95% confidence, 80% power, and 1:4 ratio, the minimum sample size came to 66:264 in the cases and controls respectively [6]. Thus, the overall minimum sample size came to 330.

**Figure 1:**
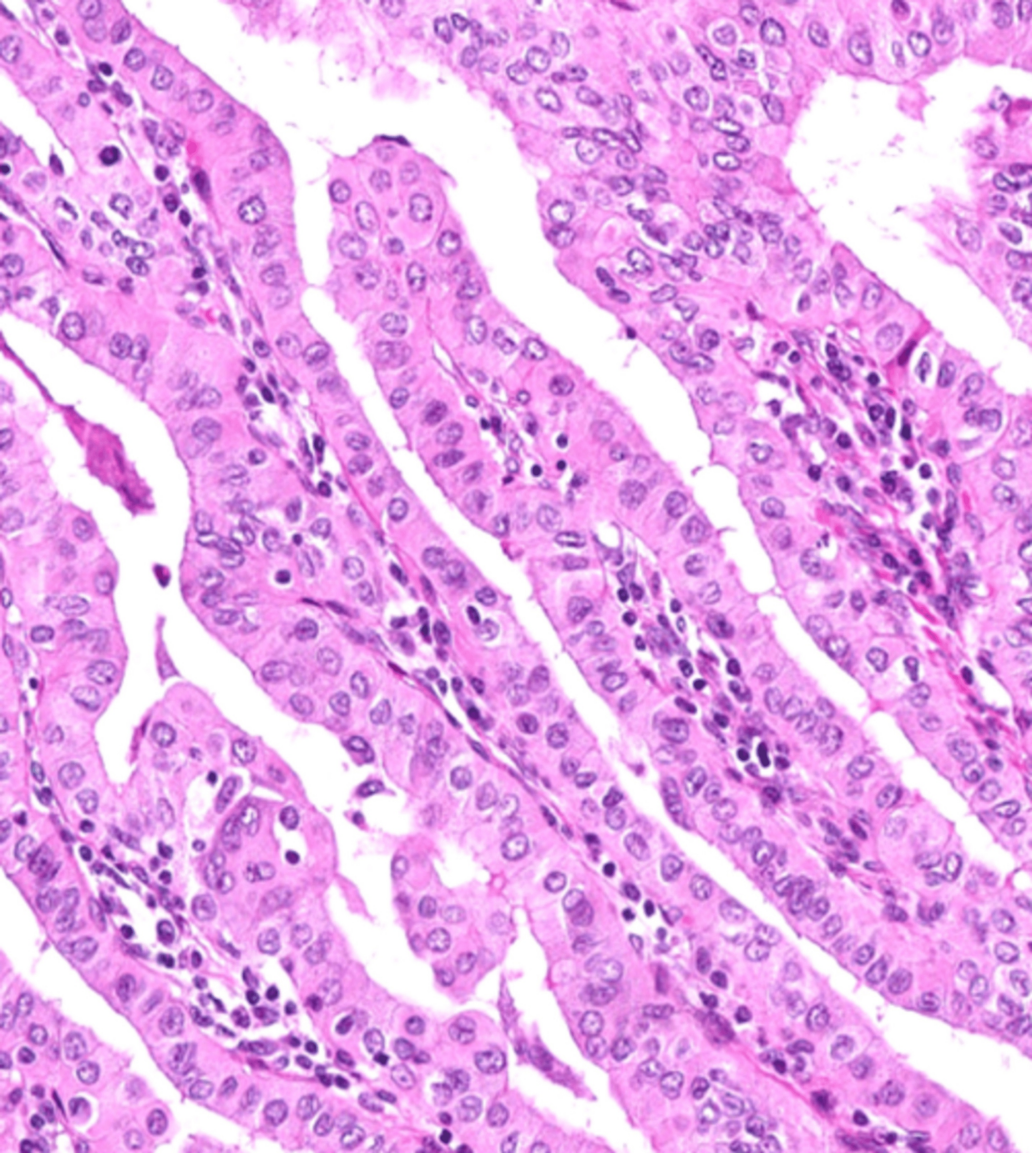
Tall cell - papillary thyroid carcinoma (PTC) with 2x-3x tall cells (H&E, 400x) A case of PTC with closely packed, elongated papillae lined by cells predominantly twice and focally thrice as tall as they are wide, with well-developed and easily identifiable nuclear features of PTC (enlarged nuclei with numerous grooves and pseudo-inclusions), sharply delineated cell borders with intensely eosinophilic cytoplasm.

**Figure 2:**
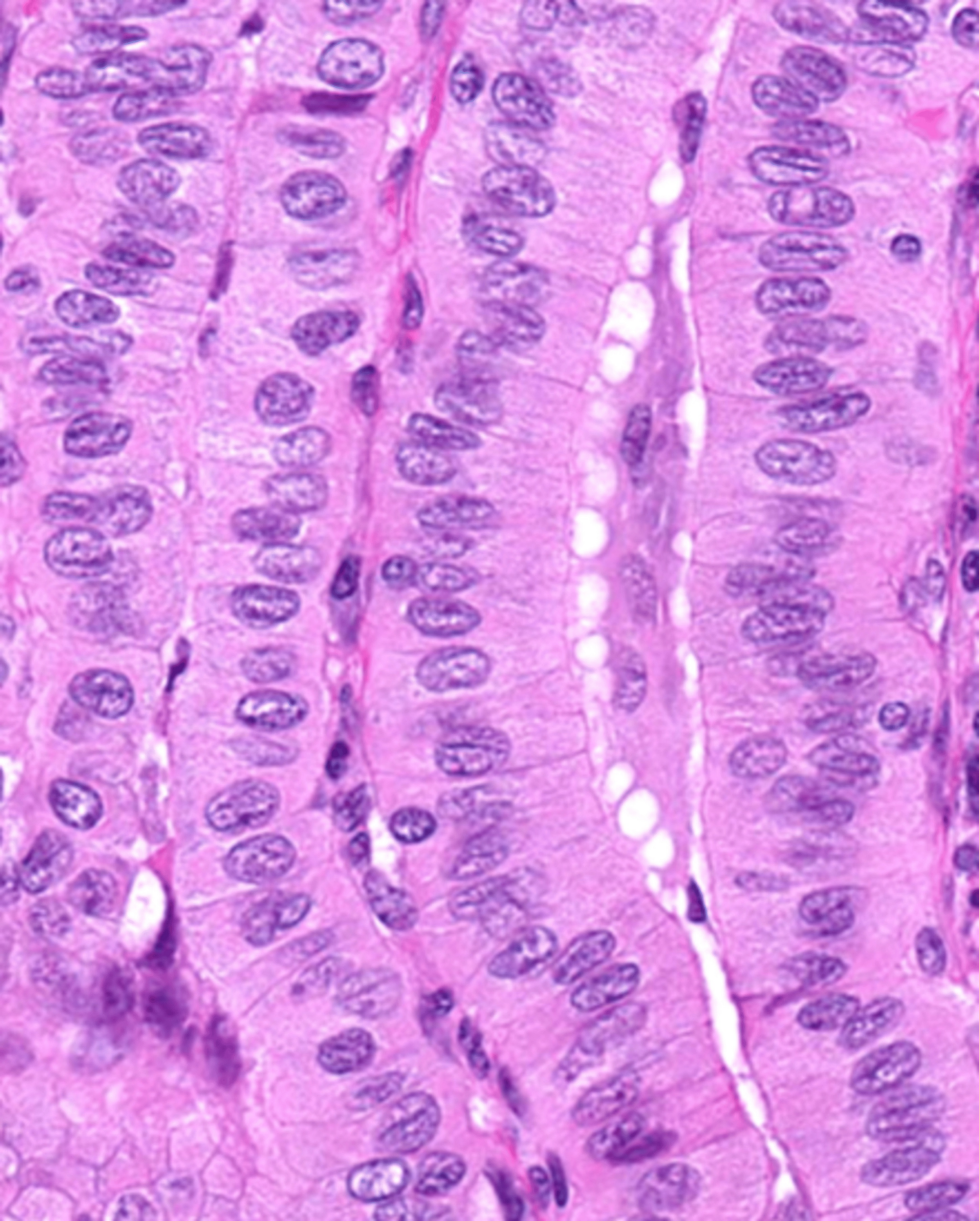
Tall cell - papillary thyroid carcinoma (PTC) with 3x tall cells (H&E, 400x) PTC with cells three times taller than they are wide, with soft histological features (abundant eosinophilic or oncocytic-like cytoplasm, distinct cell borders nuclei with exaggerated nuclear changes of PTC, and elongated "tram-track" follicles)

PTC-TCF, along with the soft histological criteria, were taken as cases (Figures 1, 2) and those with pure conventional morphology (without any tall cell component) were taken as the control group. 

Cases with mixed histological patterns involving other PTC subtypes, inadequate or missing histopathology slides, and poorly preserved tissue precluding accurate morphological evaluation were excluded from both groups.

A search of the electronic medical records (EMR) from our hospital, conducted over the study period, identified a total of 1,054 cases of histologically confirmed cases of PTC. Among these, 70 cases (6.6%) exhibited tall cell features, defined by cells measuring two to three times taller than their width along with the soft histological criteria, regardless of percentage. Of these, 66 cases (6.2%) with archived slides available for review and a minimum follow-up duration of five years were included as study cases.

Among the remaining 984 cases without tall cell component, 604 cases (61.4%) demonstrating cPTC in combination with other subtypes were excluded. From the remaining 380 cases, 264 cases (69.5%) with available archived slides and follow-up data were randomly selected as controls.

Clinical and histopathological parameters were collected from the EMR. 

Presence of locoregional recurrence of disease, distant metastasis, and deaths were recorded as follow-up data, which were obtained via the institutional cancer registry. For external referral cases, available follow-up information was retrieved from hospital records and, when feasible, verified through telephonic contact. Cases for which patients could not be contacted (n = 25, 7.6%) were classified as lost to follow-up and were excluded from outcome analysis.

Archived H&E-stained slides of cases with tall cell components were collected and reassessed for degree and percentage of tall cells. For faded slides, blocks were cut and stained fresh for the study. All the morphological analysis for the study was done by a single head and neck pathologist, although the initial diagnosis was given randomly by an additional 3 experienced pathologists earlier.

Although independent slide reviews were performed, a formal interobserver variability analysis (Kappa statistic) was not conducted. However, diagnostic discrepancies, when present, were resolved by joint review and consensus.

The study was approved by the institutional ethics committee and authorization of use of patient registry was obtained from the head of the department.

Data was analysed using IBM SPSS Statistics version 20.0 (IBM Corp., Armonk, NY, USA). Categorical variables were expressed as frequency and percentage, and associations between categorical parameters were assessed using the Chi-square test or Fisher’s exact test, as appropriate. Continuous variables were summarised as mean ± standard deviation (SD) or median with interquartile range (IQR) based on data distribution.

Normality of continuous variables was tested using the Shapiro-Wilk test and by inspecting Q-Q plots. Variables that did not conform to normal distribution were analysed using non-parametric tests. Before inclusion in multivariate models, collinearity diagnostics were performed using Variance Inflation Factor (VIF) and tolerance values to ensure absence of multicollinearity (VIF < 5 was considered acceptable).

Variables that were significant on univariate analysis (p < 0.05) were entered into the multivariate model. Model adequacy and proportional hazard assumptions were verified using log-minus-log plots and Schoenfeld residuals. A p-value < 0.05 was considered statistically significant.

## Results

All 330 cases included in the study were evaluated for the percentage and degree of tall cells on H&E-stained sections. Comparative analysis between PTC-TCF and cPTC was performed based on clinical and histopathological parameters.

The clinical parameters analysed included age, gender distribution, family history of thyroid malignancy, clinical presentation, duration of symptoms, presence or absence of pressure symptoms, thyroid hormone status, and ATA risk stratification category. The histopathological parameters included tumour focality, ETE, LVI, number of lymph nodes involved, size of the largest involved lymph node, extranodal spread, and TNM stage.

Prognostic outcomes assessed were recurrence (locoregional and/or distant) and death.

In total, 330 patients were included in the study, comprising 102 males and 228 females, with ages ranging from 12 to 81 years (median age: 44 years). The majority of PTC-TCF cases were of T3 stage (n = 21; 51.4%), while most classical PTC cases belonged to the T1 stage (n = 145; 68.5%) (Table 1).

**Table 1:** Clinical profile of the study subjects ATA - American Thyroid Association, cPTC - classical papillary thyroid carcinoma, PTC-TCF - PTC with any amount of tall cell features, MNG - multinodular goiter

Characteristic		PTC-TCF	cPTC	p-value
Gender	Male	20 (30.3%)	82 (31.1%)	0.905
	Female	46 (69.7%)	182 (68.9%)	
Age distribution	<20 years	1 (1.5%)	12 (4.5%)	0.585
	20-40 years	27 (40.9%)	112 (42.4%)	
	40-60 years	30 (45.5%)	103 (39%)	
	>60 years	8 (12.1%)	37 (14%)	
Family history of thyroid malignancy	Yes	3 (4.5%)	9 (3.5%)	0.819
	No	57 (86.3%)	200 (75.7%)	
	Unknown	6 (9.2%)	55 (20.8)	
Clinical presentation	Diffuse neck swelling	38 (57.6%)	187 (70.8%)	0.005
	Solitary thyroid nodule	14 (21.2%)	26 (9.8%)	
	MNG	11(16.7%)	17 (6.4%)	
	Lymph node enlargement	1 (1.5%)	7 (2.7%)	
	Distant metastasis	0 (0%)	2 (0.8%)	
	Asymptomatic	2 (3%)	25 (9.5%)	
Duration of symptoms	<6 months	28 (42.4%)	139 (52.7%)	0.021
	6 months - 1 year	23 (34.8%)	53 (20.1%)	
	1-5 years	14 (21.2%)	50 (18.9%)	
	>5 years	1 (1.5%)	22(8.3%)	
Pressure symptoms	Yes	9 (13.6%)	23 (8.8%)	0.609
	No	52 (78.8%)	165 (62.5%)	
	Not known	5 (7.8%)	76 (28.7)	
Thyroid hormone status	Hypothyroid	16 (24.2%)	60 (22.7%)	0.753
	Hyperthyroid	23 (34.8%)	90 (34%)	
	Euthyroid	15 (22.7%)	74 (28%)	
	Not available	12 (18.2%)	40 (15.1%)	
Modified ATA risk class	low risk and low risk-like cases	9 (13.6%)	189 (71.6%)	0.00
	Intermediate & high-risk cases	57 (86.4%)	75 (28.4%)	

Of the total 330 patients included in the study, 296 (89.7%) underwent surgery followed by radioactive iodine therapy, while the remaining 34 (10.3%) patients underwent surgery alone. The median follow-up duration was 79 months, ranging from 65 to 115 months.

The study cohort was divided into two groups based on the ATA risk stratification system. The first group comprised ATA low-risk and low-risk-like cases. ATA low-risk cases were defined as PTCs that lacked tall cell features and demonstrated no evidence of local or distant metastasis. These tumours had undergone complete macroscopic excision, showed no invasion into locoregional structures or vascular invasion, and were either clinically node-negative (N0) or had ≤5 N1 micrometastatic lymph nodes. The ATA low-risk-like category included tumours that fulfilled all the low-risk criteria except for the presence of tall cell features. The second group consisted of ATA intermediate and high-risk cases, classified according to ATA guidelines, but similarly excluding the presence of tall cells as a defining parameter

Among the 66 cases with a tall cell component, two subgroups were further analysed: tumours with 2×-3× tall cells, using cutoffs of ≥10%, ≥15%, ≥30%, and ≥50%; and tumours with 3× tall cells, using a cutoff of ≥30%.

Comparative analysis between the two groups revealed that PTC-TCF showed a significantly higher frequency of ETE into both adipose tissue and muscle (p = 0.00), higher pT stage (p = 0.00), advanced pM stage (p = 0.005), as well as increased rates of recurrence (p = 0.00) and mortality (p = 0.020) when compared to the control group (Table 2).

**Table 2:** Univariate analysis of clinicopathological parameters between papillary thyroid carcinoma with any tall cell features (PTC-TCF) and classical PTC (cPTC) ETE - extrathyroidal extension

Tumour characteristics		PTC-TCF	cPTC	P VALUE
TUMOUR FOCALITY	UNIFOCAL	28 (42.4%)	111 (42%)	0.056
	MULTIFOCAL	36 (54.5%)	82 (31%)	
	UNKNOWN	2 (3.1%)	71 (26.9%)	
EXTRATHYROIDAL EXTENSION	YES – INTO MUSCLE	14 (21.2%)	3 (1.1%)	0.00
	YES- INTO ADIPOSE TISSUE	17 (25.7%)	61 (23.1%)	
	NO ETE	32 (48.5%)	129 (48.8%)	
	INFO NOT AVAILABLE	3 (4.6%)	71 (26.9%)	
LYMPHOVASCULAR INVASION	YES	14 (21.2%)	46 (17.4%)	0.583
	NO	49 (74.2%)	133 (50.3%)	
	NOT AVAILABLE	3 (4.6%)	85 (32.2%)	
NUMBER OF LYMPH NODES INVOLVED	≤ 5	3 (4.6%)	85 (32.2%)	0.125
	>5	14 (21.2%)	32 (12.1%)	
	NONE	35 (53%)	76 (28.7%)	
	INFO NOT AVAILABLE	14 (21.2%)	71 (26.9%)	
SIZE OF THE LARGEST LYMPH NODE INVOLVED	≥3 cm	13 (19.7%)	30 (11.3%)	0.057
	<3 cm	14 (21.2%)	74 (28%)	
	NA	39 (59.1%)	160 (60%)	
EXTRANODAL SPREAD	YES	11 (16.6%)	28 (10.6%)	0.162
	NO	16 (24.2%)	76 (28.7%)	
	NA	39 (59.2%)	160 (60%)	
pT STAGE	T1a	10 (15.1%)	114 (43.2%)	0.00
	T1b	21 (31.8%)	31 (11.7%)	
	T2	14 (21.2%)	52 (19.7%)	
	T3a	10 (15.1%)	41 (15.5%)	
	T3b	11 (16.6%)	22 (8.3%)	
	T4	0 (0%)	4 (1.5%)	
pN STAGE	Nx	24 (36.3%)	138 (52.2%)	0.077
	N0	15 (22.7%)	43 (16.3%)	
	N1a	11 (16.6%)	42 (15.9%)	
	N1b	16 (24.2%)	41 (15.5%)	
pM STAGE	M0	13 (19.7%)	13 (4.9%)	0.005
	M1	16 (24.2%)	14 (5.3%)	
	Mx	37 (56%)	237 (89.7%)	
RECURRENCE ON FOLLOW-UP	LOCOREGIONAL	10 (15.1%)	7 (2.6%)	0.00
	DISTANT	2 (3%)	2 (0.7%)	
	NONE	54 (81.8%)	230 (87.1%)	
	NOT KNOWN	0 (0 %)	25 (9.5%)	
DEATHS	YES	2 (3%)	3 (1.1%)	0.02
	NO	64 (96.9%)	236 (89.4%)	
	NOT KNOWN	0 (0 %)	25 (9.5%)	

During follow-up, nine recurrences (n=9; 3.4%) were observed in the cPTC cohort, whereas 12 recurrences (n=12; 18.2%) occurred in the PTC-TCF cohort, a difference that was statistically significant (p = 0.00).

A total of three deaths (n=3; 1.13%) were recorded in the cPTC group, compared to two deaths (n=2; 3%) in the PTC-TCF group, which also reached statistical significance (p = 0.020).

On performing multivariate analysis of the clinicopathological parameters that showed statistical significance between the two groups, after adjusting for tumour size and nodal metastasis, only the T2 (p = 0.00, HR = 12.697) and T3 (p = 0.00, HR = 17.395) stages retained independent prognostic significance (Table 3).

**Table 3:** Multivariate analysis of clinicopathological parameters between papillary thyroid carcinoma with any tall cell features (PTC-TCF) and classical PTC (cPTC) CI - confidence interval

VARIABLE	P-VALUE	HAZARD RATIO	95% CI	
			UPPER	LOWER
T2	0.00	12.697	4.838	33.320
T3	0.00	17.395	7.057	42.877

On univariate analysis of the various clinicopathological parameters with the incidence of recurrence and mortality, it was found that tumour type, tumour size, modified ATA risk class, proportion of 2x cells (with 30% cutoff), T stage, and M stage were statistically significant (with p-values of 0.00, 0.043, 0.006, 0.003, 0.043, and 0.021 respectively) (Table 4).

**Table 4:** Univariate analysis of clinicopathological parameters vs any event (recurrence or mortality) PTC -  papillary thyroid carcinoma

Tumor characteristics		ANY EVENT PRESENT	NO EVENTS	p- VALUE
TUMOUR TYPE	CLASSICAL PTC	12 (4.5%)	252 (95.5%)	0.000
	TALL CELL SUBTYPE OF PTC	11 (21.5%)	40 (78.5%)	
	CLASSICAL PTC WITH TALL CELL AREAS	3 (20%)	12 (80%)	
ATA RISK CLASS	LOW	7 (5.5%)	119 (94.5%)	0.120
	INTERMEDIATE	7 (7.6%)	85 (92.4%)	
	HIGH	12 (10.7%)	100 (89.3%)	
PROPORTION OF 2x CELLS	<30%	17 (6%)	264 (94%)	0.003
	≥30%	9 (18.3%)	40 (81.7%)	
PROPORTION OF 2x CELLS	<50%	21 (7.1%)	272 (92.9%)	0 .177
	≥50%	5 (13.5%)	32 (86.5%)	
PROPORTION OF 3x CELLS	<30%	24 (7.5%)	295 (92.5%)	0.197
	≥30%	2 (18.2%)	9 (81.8%)	
PROPORTION OF 2x CELLS	<10%	21 (7.9%)	245 (92.1%)	
	≥10%	5 (7.8%)	59 (92.2%)	
PROPORTION OF 2x CELLS	<15%	17 (6%)	264 (94%)	
	≥15%	9 (18.3%)	40 (81.7%)	
TUMOUR SIZE	LESS THAN OR EQUAL TO 1CM	6 (11.5%)	46 (88.5%)	0.043
	>1CM & ≤2CM	5 (3.5%)	134 (96.5%)	
	>2CM & ≤4CM	4 (6.7%)	54 (93.3%)	
	>4CM	11 (13.5%)	70 (86.5%)	
TUMOUR FOCALITY	UNIFOCAL	12 (8.6%)	127 (91.4%)	0.580
	MUTLIFOCAL	8 (6.7%)	110 (93.3%)	
	UNKNOWN	6 (8.2%)	67 (91.8%)	
EXTRATHYROIDAL EXTENSION (ETE)	YES- INTO MUSCLE	1 (5.8%)	16 (94.2%)	0.204
	YES- INTO ADIPOSE TISSUE	10 (12.8%)	68 (87.2%)	
	NO ETE	10 (6.2%)	151 (93.8%)	
	INFO NOT AVAILABLE	5 (6.7%)	69 (93.3%)	
LYMPHOVASCULAR INVASION	YES	6 (10%)	54 (90%)	0.573
	NO	14 (7.7%)	168 (92.3%)	
	NOT AVAILABLE	6 (6.8%)	82 (93.2%)	
NUMBER OF LYMPH NODES INVOLVED	LESS THAN OR EQUAL TO 5	5 (5.8%)	80 (94.2%)	0.771
	MORE THAN 5	3 (6.5%)	43 (93.5%)	
	NONE	10 (8.7%)	104 (91.3%)	
	INFO NOT AVAILABLE	8 (9.4%)	77 (90.6%)	
SIZE OF THE LARGEST LYMPH NODE INVOLVED	>3CM	3 (6.9%)	40 (93.1%)	0.721
	<3CM	5 (5.6%)	83 (94.4%)	
	NA	18 (9%)	181 (91%)	
PERINODAL SPREAD	YES	2 (5%)	37 (95%)	0.671
	NO	6 (6.5%)	86 (93.5%)	
	NA	18 (9%)	181 (91%)	
‘T’ STAGE OF TNM	T1a	10 (7%)	131 (93%)	0. 021
	T1b	1 (1.9%)	51 (98.1%)	
	T2	4 (7.1%)	52 (92.9%)	
	T3a	10 (19.6%)	41 (80.4%)	
	T3b	1 (4%)	24 (96%)	
	T4	0 (0%)	5 (100%)	
‘N’ STAGE OF TNM	Nx	14 (7.3%)	178 (92.7%)	0.274
	N0	5 (10.4%)	43 (89.6%)	
	N1a	1 (2.3%)	42 (97.7%)	
	N1b	6 (12.7%)	41 (87.3%)	
‘M’ STAGE OF TNM	M0	3 (18.7%)	13 (81.3%)	0.014
	M1	4 (22.2%)	14 (77.8%)	
	Mx	19 (6.4%)	277 (93.6%)	

On performing multivariate analysis of these parameters after controlling for tumour focality, ETE, and LVI, the parameter of 2x tall cell criteria with a 30% cutoff was solely found to retain significance with regards to prognosis (p-value = 0.048 and Hazard ratio = 2.46 (Table 5)). 

**Table 5:** Multivariate analysis of clinicopathological parameters vs. any event (recurrence/mortality) CI - confidence interval

VARIABLE	P-VALUE	HAZARD RATIO	95% CI	
			UPPER	LOWER
Proportion of 2x tall cells (<30% & ≥30%)	0.048	2.462	0.969	6.257

## Discussion

Overview and historical context

PTC is the most prevalent thyroid malignancy, and the tall cell subtype is widely recognized for its aggressive biological behaviour. First described by Hawk and Hazard in 1976, tall cells were originally defined as being twice as tall as they are wide, a ratio that later evolved to 3:1 in the 2004 WHO classification [7]. Since then, both 2:1 and 3:1 ratios have been variably applied, along with differing thresholds for tall cell proportion (30-100%), resulting in inconsistent diagnostic criteria [3].

The WHO 2022 classification now defines TC-PTC as PTCs containing ≥30% of cells that are at least three times as tall as they are wide and show dense eosinophilic cytoplasm and distinct cell membranes [4]. However, the clinical implications of this stricter definition remain uncertain, and its prognostic relevance is still under investigation.

Key findings and implications of the WHO 2022 criteria

In our cohort of 66 cases with tall cell features, the presence of 2x tall cells in ≥30% of the tumour was the only clinicopathological variable that remained independently predictive of recurrence and mortality (p = 0.048, HR = 2.46) after adjusting for tumour focality, ETE and LVI. This indicates that a broader morphologic definition has stronger prognostic relevance than the WHO 2022 criterion. One plausible technical explanation for this would be an oblique tissue sectioning through variably aligned papillae underestimating true cell height, thus leading to underrepresentation of 3× tall cells on routine histology in any given case. Keeping a flexible criterion of 2x tall cells with tumour focally attaining 3x tall cell criteria along with soft histological features might have turned more robust in addressing this underestimation. 

When the WHO 2022 threshold (≥30% of 3× tall cells) was applied strictly, only 11 of 66 PTC-TCFs qualified as TC-PTC, and frequency of outcomes in this subset were not statistically significant (p = 0.197). This supports the conclusion that the WHO 2022 definition may underdiagnose biologically aggressive cases.

Comparison with previous studies

Our findings differ from those of Turchini et al., who observed significant correlations between tall cell morphology and poor prognosis using stricter thresholds (2× 50%, 3× 30%, 3× 50%; p = 0.049, 0.004, 0.004, respectively), whereas their 2× 30% cutoff was not significant (p = 0.063) [5]. These differences likely stem from our smaller sample size and our inclusion of soft histological features in the diagnostic criteria as well. 

Clinicopathological correlation

Our results clearly demonstrate the aggressive phenotype of PTC-TCFs compared with cPTCs. Statistically significant differences were observed for tumour size >2 cm (81.5% in PTC-TCF, p = 0.001), multifocality (54.5%), and ETE (21.2%, p < 0.001). These findings align with previous studies describing similar aggressive histopathologic features [8-11].

PTC-TCFs also showed higher frequencies of symptomatic solitary nodules and multinodular goitre (p = 0.005), and shorter symptom duration (<5 years), consistent with rapid progression. Despite similar overall metastasis rates at diagnosis (27.3% in PTC-TCF vs 28% in cPTC), site distribution differed significantly (p = 0.001), with tall cell cases more often involving mediastinal nodes, lungs, and bones.

Furthermore, advanced tumour stages were strongly associated with PTC-TCFs: T2/T3 stage in 80.2% (p < 0.001) vs 43.5% in cPTC, and M1 stage in 12.1% vs 3.8% in cPTC (p = 0.005).

Multivariate analysis reinforced that only T2 (HR = 12.70, p < 0.001) and T3 (HR = 17.40, p < 0.001) stages were consistently associated with PTC-TCF over cPTC.

Prognostic outcomes

The prognostic impact of tall cell morphology was confirmed by follow-up outcomes. Recurrence occurred in 18.2% of PTC-TCF versus 3.4% of cPTCs (p < 0.001), and mortality was higher in PTC-TCF (3.0%) than in cPTC (1.1%) (p = 0.020).

These statistically significant differences highlight that tall cell morphology, even in partial proportion, portends a worse clinical course. These results are consistent with previous studies by Longheu et al. and Taibo et al., which reported similarly elevated recurrence and mortality rates in tall cell variants [12,13].

Interpretation of atypical findings

Although the PTC-TCF group demonstrated more aggressive biological features overall, certain parameters in our study - such as the proportion of cases with clinically enlarged lymph nodes, distant metastasis, and disease duration exceeding five years - were unexpectedly higher among cPTCs. This apparent discrepancy may be explained by several factors.

As cPTCs with overall indolent biology tend to remain for longer durations before presenting itself, the likelihood of nodal or distant involvement at presentation is also found to be more. In contrast, PTC-TCFs tend to present earlier with high-risk features, which could limit the number of patients presenting with distant and nodal metastasis.

Lymph node enlargement at the time of clinical examination in classical PTCs may reflect reactive hyperplasia or micrometastasis rather than true aggressive nodal disease, whereas in PTC-TCFs, metastatic nodes are typically smaller but histologically more infiltrative [1,3].

Differences in tumour biology and pattern of spread may also play a role, as cPTCs tend to metastasize via lymphatic channels, while tall cell variants have a higher predilection for local invasion and direct extension.

## Conclusions

Papillary thyroid carcinoma with tall cell features represents a distinct and biologically aggressive variant within the PTC spectrum. Our results demonstrate that diagnosis with a tall cell criterion using a broader morphologic threshold, cells two to three times as tall as wide in ≥30% of the tumour along with soft morphological features endorsed by WHO - significantly associated with poor prognostic outcomes (p = 0.048, HR = 2.46), whereas the WHO 2022 definition (≥30% of 3× tall cells) was not able to predict outcomes (p = 0.197).

PTC-TCFs were characterized by larger tumour size (p = 0.001), higher multifocality, ETE (p < 0.001), and advanced stage (T2/T3, p < 0.001), along with increased recurrence (p < 0.001) and mortality (p = 0.020). These findings reinforce that tall cell morphology is a robust adverse prognostic factor, even when present in smaller proportions.

While the WHO 2022 criteria provide diagnostic uniformity, they may exclude a subset of clinically aggressive tumours. Adopting a broader morphological definition could improve risk stratification and prognostic accuracy. Nevertheless, due to the retrospective design and missing data in a minority of cases, causal inference should be made with caution, and validation through prospective multicentric studies is warranted.
